# Case report: Re-evaluating reversibility of cytotoxic lesions of the corpus callosum

**DOI:** 10.3389/fnimg.2025.1436931

**Published:** 2025-02-17

**Authors:** Victoria Vold, Stein-Helge Hansen Tingvoll, Mona K. Beyer, Kaja Nordengen

**Affiliations:** ^1^Institute of Clinical Medicine, Faculty of Medicine, University of Oslo, Oslo, Norway; ^2^Department of Neurology, Lillehammer Hospital, Lillehammer, Norway; ^3^Ringen Rehabilitation Center, Moelv, Norway; ^4^Division of Radiology and Nuclear Medicine, Oslo University Hospital, Oslo, Norway; ^5^Department of Neurology, Oslo University Hospital, Oslo, Norway

**Keywords:** case report, cytotoxic edema, corpus callosum, centrum semiovale, MRI

## Abstract

Cytotoxic lesions of the corpus callosum (CLOCC) are a clinicoradiological diagnosis, characterized by transient neurological symptoms and magnetic resonance imaging (MRI) changes in the splenium of the corpus callosum (SCC), which in most cases is completely reversible. However, the long-term pathophysiological trajectory and ultimate neurological outcomes of CLOCC remain largely unknown due to limited long-term follow-up data. We report an 11-year follow-up of a postpartum female with CLOCC, initially presenting with transient focal neurological symptoms and extensive diffusion-restricted white matter involvement including the SCC and surrounding area with diffusion restriction and low apparent diffusion coefficient values, indicative of cytotoxic edema. The edema regressed in days; over the years, she remained asymptomatic despite persistent white matter changes on MRI in the centrum semiovale. This case challenges the view of CLOCC as completely reversible and raises questions regarding the significance of lasting white matter changes. The enduring absence of neurological symptoms and stable radiological profile throughout the decade underscores the singular nature of CLOCC and the lasting, though isolated, impact on white matter. This report contributes a crucial perspective, suggesting that CLOCC may involve just an isolated episode without recurrent events or progressive neurological decline. By offering the first longitudinal analysis of a CLOCC episode with an extended follow-up of over a decade, our case enhances current knowledge about the long-term neurological and radiological landscape of this condition. It suggests a reevaluation of the conceptual understanding of CLOCC as an entirely reversible, non-relapsing disorder, highlighting the need for further research into its long-term impacts on cerebral white matter integrity.

## Introduction

Cerebral edema, describing swelling of brain tissue (Nehring et al., 2024), can be categorized into cytotoxic, vasogenic, interstitial, and osmotic edema (Nehring et al., 2024), all of which display high signal on T2 weighed and low signal on T1 weighed sequences on magnetic resonance imaging (MRI; Ho et al., 2012). Cytotoxic edema is intracellular fluid buildup in neurons and/or glial cells due to ischemic or excitotoxic cerebral injury, almost always with subsequent cellular death (Ho et al., 2012; Moritani et al., 2005; de Oliveira et al., 2019). On MRI, it is characterized by decreased diffusion on diffusion-weighed imaging (DWI), resulting in a low apparent diffusion coefficient (ADC; Ho et al., 2012). This represents the decreased movement of extracellular fluid due to it shifting into the intracellular compartment, as well as restricted diffusion of water molecules over the cell membranes and in the cytoplasm of the affected cells (Ho et al., 2012; Al Brashdi and Albayram, 2015).

While cytotoxic edema generally leads to cell death, the phenomenon of cytotoxic lesions of the corpus callosum (CLOCC) challenges conventional understandings, particularly due to its reversible nature, contrasting the typical outcomes associated with cytotoxic edema. Since 1996, 480 English reports of cases with the same radiological characteristics of reversible lesions in the splenium of the corpus callosum (SCC), showing signs of cytotoxic edema, with concurrent neurological symptoms, have been published (Chason et al., 1996; Moors et al., 2023). The lesions are generally oval, homogenous, non-hemorrhagic, and non-enhancing, centrally placed in the SCC, and occasionally involve the surrounding area (Barburoglu et al., 2022). The MRI characteristics are very well aligned, with all lesions being hyperintense on DWI, slightly hyperintense on T2- and fluid attenuated inversion recovery (FLAIR)-sequences, hypointense in T1-sequences, with decreased ADC values, and without contrast enhancement (Barburoglu et al., 2022; Tetsuka, 2019; Blaauw and Meiners, 2020), all indicative of cytotoxic edema (Ho et al., 2012; Tetsuka, 2019). The above-mentioned surprising reversibility normally manifests within 1–2 weeks (Moors et al., 2023; Garcia-Monco et al., 2011). However, comprehensive long-term follow-up data is absent. Addressing these gaps could provide valuable insights into whether the changes consistently reported are entirely benign or if they mask more subtle, lasting effects on brain health.

Reported symptoms show a larger variability, but most display a generalized rather than focal picture, including headache, cognitive impairment, seizures, behavior changes, drowsiness, confusion, general motor deterioration, delirium, somnolence, dizziness, disconnection syndrome, hallucinations, and coma (Tetsuka, 2019; Blaauw and Meiners, 2020). However, some report dysarthria, tremor, ataxia and visual disturbance, consistent with a posterior affection rather than generalized (Tetsuka, 2019; Blaauw and Meiners, 2020). The symptoms typically disappear completely within a few days to a month (Tetsuka, 2019; Aksu Uzunhan et al., 2021), but timeframes down to a few hours have been reported (Park et al., 2013). Among adults, the median age of CLOCC is 37 years (interquartile range 24–46 years), and there are no sex differences (52% males, 47% females, and 1% unknown sex; Moors et al., 2023).

Several conditions have been revealed as possible causes of CLOCC. They include toxin exposure or drug withdrawal (27%), viral infections (18%), bacterial or plasmodia infections (10%), vascular episodes (18%), seizures (6%), and metabolic disturbances (3%; Moors et al., 2023; Tetsuka, 2019). 15 peri- and postpartum transient callosal lesions (Chen et al., 2012; Oliveira and Zaidat, 2014; Altunkas et al., 2016; Curtis et al., 2013; Shah and Little, 2020; Ueda et al., 2014; Takahashi et al., 2014; Liu et al., 2017; Udaya et al., 2015; Hiraga et al., 2016; Sekine et al., 2012; Suzuki et al., 2023; Yang et al., 2019; Saif et al., 2021), as well as several cases with no identifiable cause, have also been described (Moors et al., 2023). The pathogenesis of CLOCC is still unclear, but the most accepted theory is inflammatory cytokinopathy in the brain causing excitotoxicity, resulting in cytotoxic edema and therefore restricted diffusion (Moors et al., 2023; Barburoglu et al., 2022). The SCC might be especially susceptible to this effect due to its high blood flow supplied from both the anterior and posterior circulation (Tetsuka, 2019). Studies have suggested that the corpus callosum has a high density of glutamate receptors and aquaporin type 4 channels (Hassel et al., 2003; Goursaud et al., 2009; Badaut et al., 2014), potentially also contributing to this susceptibility.

During recent years we have gained a greater understanding of CLOCC's pathogenesis, etiology, and clinical and radiological presentation, largely due to reports describing individual cases. However, no publications have described its long-term development and prognosis. In this report, we present the first case of a patient with findings that fit the description of CLOCC with a follow-up period spanning over a decade.

## Patient information

A previously healthy 24-year-old Norwegian woman woke up in the morning with paresis in her right arm and dysarthria, which resolved spontaneously after 2 h. This occurred 10 days after a normal vaginal delivery of a healthy child without any complications during pregnancy. She was receiving treatment with dicloxacillin for a mastitis diagnosed 2 days prior, where laboratory examinations had indicated elevated leukocyte counts (17.3 × 10^9^/L). Additionally, she had experienced a right frontal pulsating headache the preceding 2 days, rated 3 out of 10 at a numerical rating scale, subsequently decreasing in intensity. The patient denied any history of heritable diseases, substance abuse, episodes of confusion, trauma, or convulsions. Throughout her pregnancy, blood pressure and urine test strips performed in accordance with Norwegian guidelines for antenatal care revealed no indication of preeclampsia or gestational diabetes. Nothing in the patient's clinical history, including her job as a kindergarten teacher, suggested exposure to potentially neurotoxic agencies. Confirmatory tests for substances correlated with toxic leukoencephalopathy were therefore not conducted.

**Figure 1:**
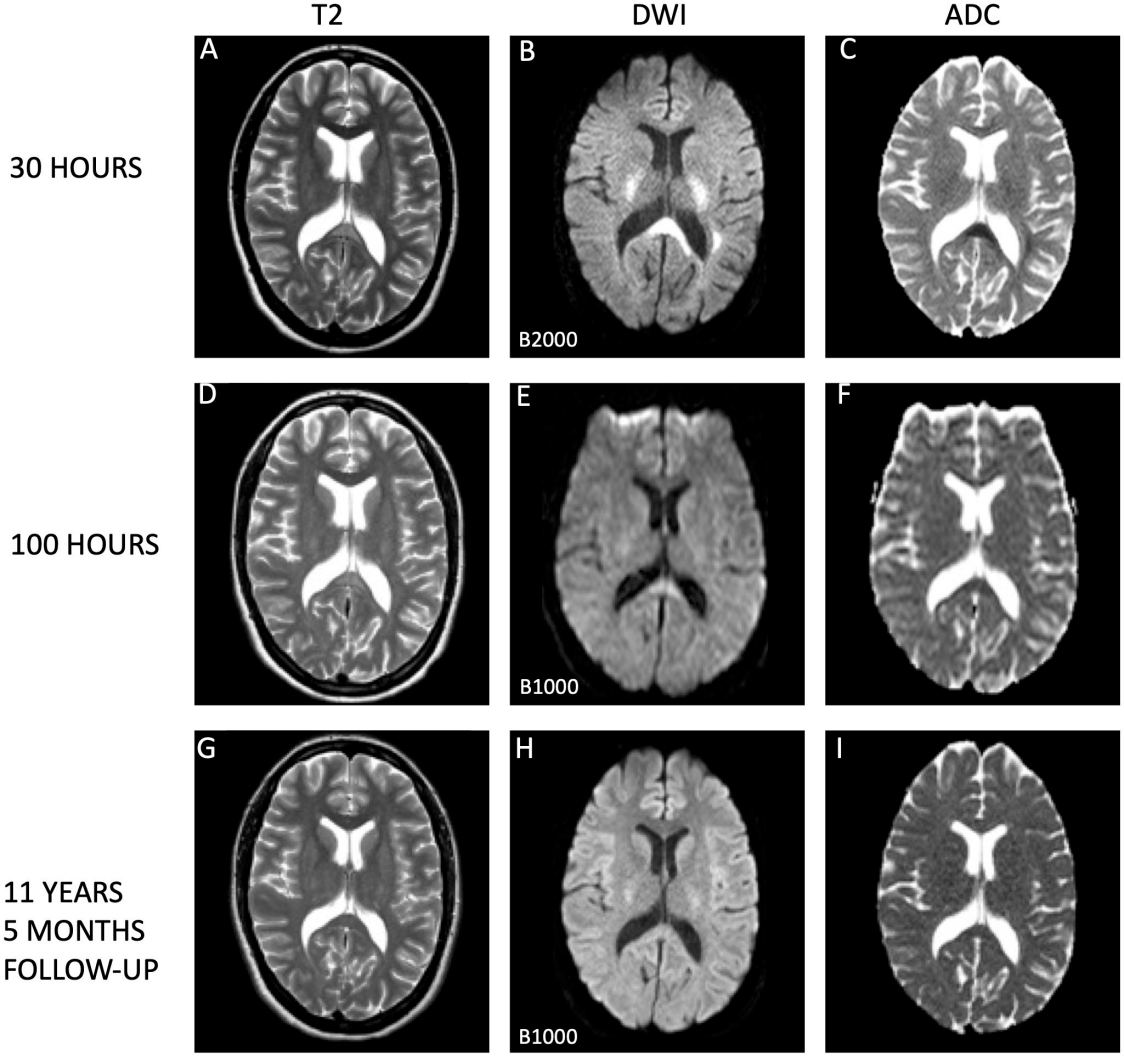
Evolution of cytotoxic edema in the splenium of the corpus callosum. This series of magnetic resonance imaging (MRI) scans tracks the progression of the splenial lesion from the acute phase to complete resolution. **(A–C)** Depict the initial scan taken 30 h after symptom onset, which shows a conspicuous lesion on T2-weighted images **(A)**, with diffusion restriction on diffusion-weighted imaging (DWI; **B**), and corresponding low apparent diffusion coefficient (ADC) values **(C)**, consistent with cytotoxic edema By 100 h post-symptom onset, scans **(D–F)** illustrate a marked diminution of hyperintensities on T2 **(D)** and a notable reduction in diffusion restriction **(E)**, accompanied by normalization of ADC values **(F)**, indication substantial lesion recovery. Long-term follow-up at 11 years and 5 months later **(G–I)** confirms complete normalization of the splenial lesion, with standard appearances on T2 **(G)**, DWI **(H)**, and ADC maps **(I)**. All MRI scans were performed on 1.5 Tesla Philips scanners: Achieva for the initial and follow-up scans **(A–C, G–I)** and Gyroscan Intera for the intermediate scans **(D–F)**. Notably, the DWI scans on day 2 utilized a B-value of B2000, in contrast to the standard B1000 used in the subsequent scans on day 5 and at long-term follow-up, which may affect the intensity of the diffusion signal. Higher B-values reflects the strength and timing of the gradients that are applied to generate DWI images. The higher the b-value, the stronger the diffusion effects. Higher b-values corresponds to images with less signal where water in tissues diffuses more rapidly. High b-values are e.g., used for detection of even small lesions, which can increase sensitivity to diffusion, but may also lead to reduced signal-to-noise ratio. Typically, values between b1000 and b2000 are used in routine diagnostic imaging. This visual depiction showcases the natural history of a splenial lesion undergoing complete resolution over an extended timeframe.

**Figure 2:**
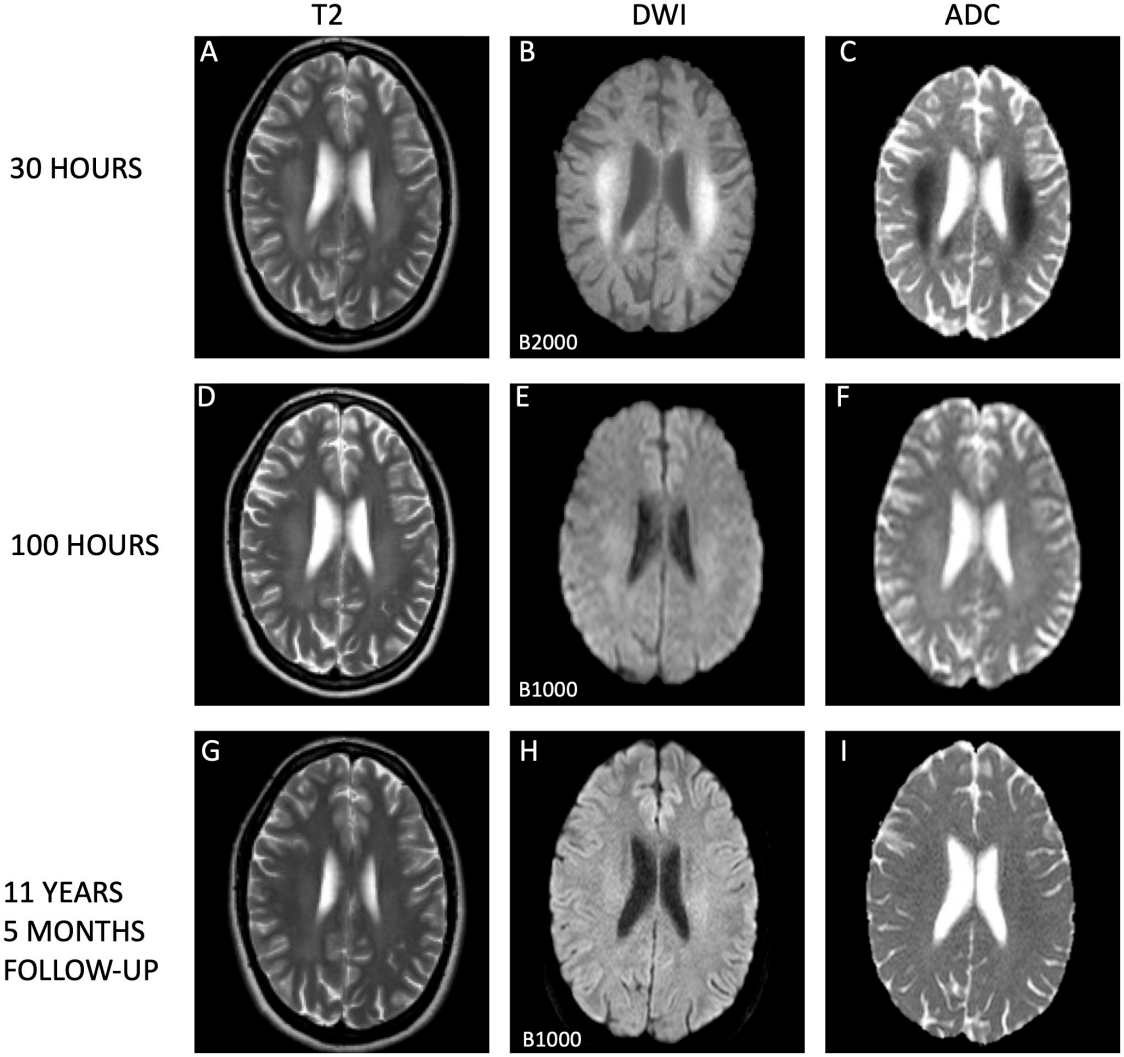
Temporal evolution of extrasplenial lesions in corona radiata. The dynamic changes in cytotoxic edema observed on magnetic resonance imaging (MRI) sequences are illustrated. Initial MRI scans **(A–C)** conducted 30 h post-symptom onset show extensive and confluent hyperintensities within the centrum semiovale on T2-weighted images **(A)** and evidence of diffusion restriction on diffusion weighted imaging (DWI; **B**), with corresponding low apparent diffusion coefficient (ADC) values **(C)**, supporting the presence of acute cutotoxic edema. Subsequent MRI scans at 100 h **(D–F, I)** display residual hyperintensities in the corona radiata **(D)** and ongoing diffusion restriction **(E)**, with normalized ADC measurements **(F)**, indicating the beginning of edema resolution. Long-term follow-up MRI scans after 11 years and 5 months reveal subtle persistent high T2 signal within the corona radiata **(G)** and on DWI **(H)**, yet ADC values have normalized **(I)**, suggesting stability of the lesions. All scans were acquired using 1.5 Tesla Philips MRI scanners, with initial and follow-up images **(A–C, G–I)** from an Achieva system and the 100-h scans **(D–F)** from a Gyroscan Intera system. It is important to note that the first DWI scans **(B)** utilized a higher B-values (B2000) compared to those from day 5 and the long-term follow-up (B1000), potentially influencing signal intensity.

The patient described being fully conscious and oriented upon waking up and noticing the symptoms. Upon arrival at the hospital approximately 2 h later, she had a Glasgow Coma Score (GCS) score of 15, which she maintained throughout the subsequent hospitalization. There were no notable systemic or neurological abnormalities upon examinations. Cognitive function tests were therefore not conducted. Blood pressure levels were slightly elevated (147/92). Blood test results revealed normalized leukocyte counts at 6.9 × 10^9^/L, but indicated high levels of infection markers, including a C-reactive protein (CRP) concentration of 70 mg/L and a sedimentation rate (SR) of 33 mm/h. Conventional computer tomography (CT) and CT angiography revealed no pathology, except a 7 × 2 mm calcification in the left internal carotid artery. MRI was performed about 30 h after the patient became aware of the symptoms. The T2 sequences revealed subtle hyperintense changes in the SCC (Figure 1), with bilateral spreading posteriorly and cranially to periventricular regions and up to centra semiovalia (Figure 2), and, to a lesser degree, down along the corticospinal tract. More surprisingly, there were diffusion restriction (Figures 1B, 2B) in the same area. The lesion demonstrated low ADC-values of 240 × 10^−3^ mm^2^/s (Figures 1C, 2C). Similar characteristics were seen in centrum semiovale, although less pronounced (Figures 2A–C).

On day three, a urine test strip confirmed the presence of blood (3+), protein (1+), and leukocytes (3+), but due to absence of symptoms indicative of a urinary tract infection, the patient's treatment with dicloxacillin for mastitis was not substituted with an antibiotic that covers common urinal tract infection pathogens. By day four, laboratory examinations indicated normalized infection markers. Lumbar puncture results on day four exhibited no signs of inflammation, with no pleocytosis, normal protein and protein ratio, normal glucose and glucose ratio, and identical oligoclonal bands in cerebrospinal fluid (CSF) and serum. Upon conducting a control MRI approximately 70 h after the initial MRI, there were persistent white matter hyperintensities in the aforementioned regions evident in the T2-weighted sequence (Figure 1D), but significant resolution in the DWI (Figure 1E) without the low ADC values from earlier, now being 686 × 10^−3^ mm^2^/s in SCC (Figure 1F). A similar normalization was seen in centrum semiovale (Figures 2D–F).

The patient stayed in hospital for 5 days for observation and assessment, with a follow-up 12 days after being discharged. Approximately 1.5 years later, the patient successfully gave birth to her second child without experiencing any neurological symptoms during or after the pregnancy.

**Figure 3:**
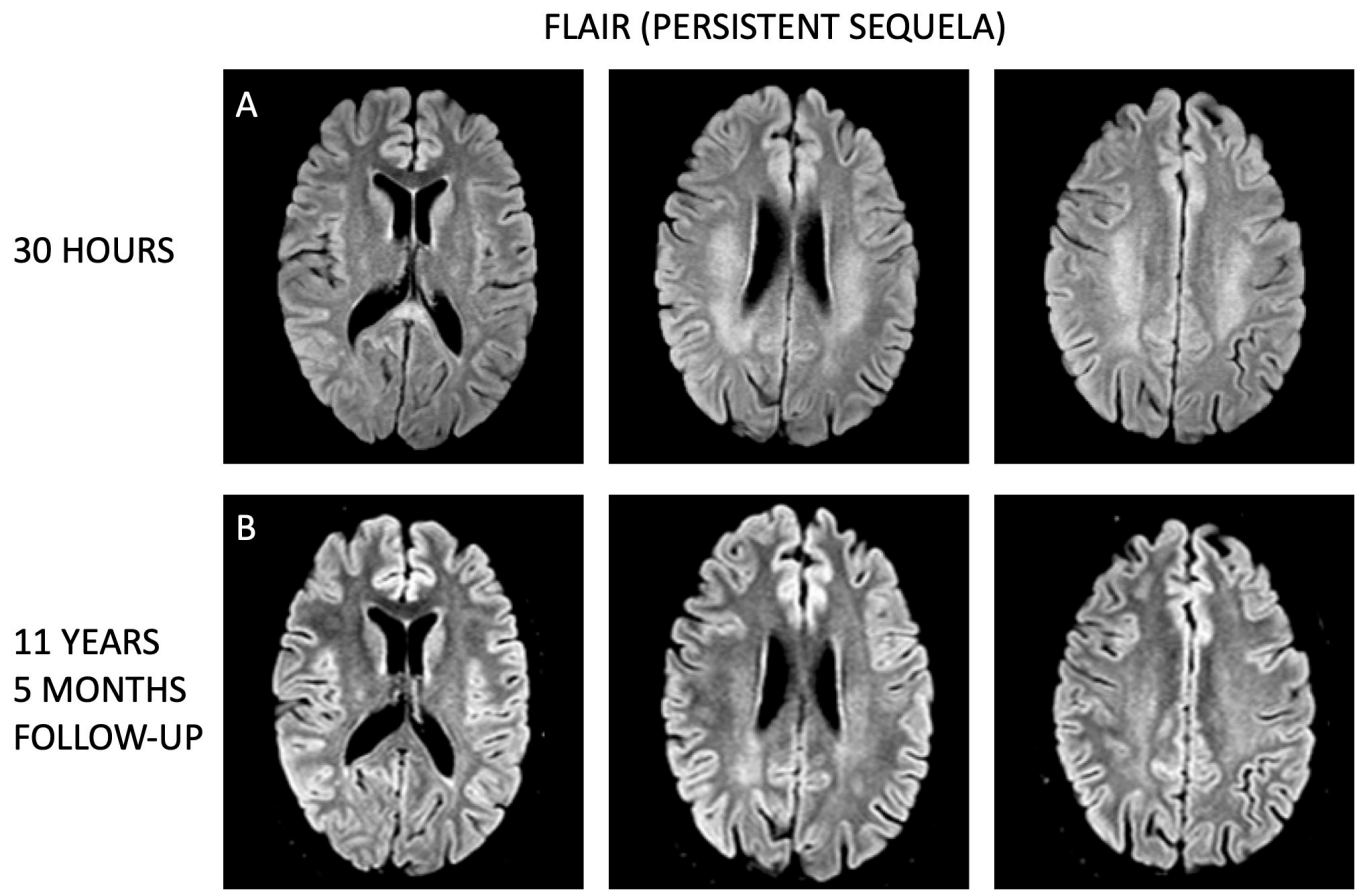
Longitudinal comparison of white matter lesions on fluid attenuated inversion recovery (FLAIR) MRI sequences. **(A)** Display axial sections from the initial MRI taken 1 day after the transient neurological symptoms, while **(B)** show corresponding axial sections from the follow-up MRI conducted 11 years and 5 months later. Despite the time gap, the white matter lesions remain visible more than a decade after, although less prominent.

In an 11-year follow-up consultation, the patient reported the absence of any new neurological symptoms or sequelae since the initial episode. The MRI showed complete resolution of the SCC lesion (Figures 1G–I), but still discreet high signal changes in the corona radiata and around the dorsal areas of the lateral ventricles in T2 (Figure 2G) and DWI (Figure 2H), with no changes in ADC values (Figure 2I). No new changes, including bleeding, calcifications, tumors, focal ischemic damage, or other focal changes in the white matter, were observed. The intracranial arteries appeared normal. A herniation of liquor within the sella turcica with a protruding adenohypophysis was detected, but considered an incidental finding. The long-term imaging sequela was most apparent in FLAIR, comparing the first MRI scan the day after the transient neurological symptoms (Figure 3A) and the recent follow-up more than 11 years later (Figure 3B), again with a complete resolution of the SCC lesion. Technical details on all three MRI scans is available in Supplementary Table, while comparative FLAIR from the first and last scan with adjusted to identical slice thickness and spacing to enhance comparability across the time points is available in Supplementary Figure.

## Discussion

CLOCC is a condition that only recently has gained more attention, with increasingly more cases and reviews being published each year. In our case report, we have presented the first decade-long follow-up of a CLOCC case. Importantly, despite being among the cases with the most widespread cytotoxic edema, there were neither radiological nor clinical reoccurrences, which is important to note to other CLOCC patients.

The patient initially presented with transient neurological symptoms, and MRI showed typical signal intensities for CLOCC on DWI, T2, FLAIR and T1 sequences, as well as low ADC-values, and near complete resolution of the MRI findings 4 days later. However, no MRI with gadolinium contrast was conducted, therefore it is uncertain whether the lesion would show the characteristic absence of contrast enhancement. Gadolinium-enhanced MRI can be crucial in delineating lesion characteristics, particularly by highlighting vascular and inflammatory responses. Although normal findings with CT angiography and the absence of inflammation in the spinal tap suggest that contrast enhancement was unlikely, the role of gadolinium-enhanced imaging in providing additional confirmation of these findings could not be explored. The patient reported no clinical sequelae or other neurological symptoms in the following 11 years, and follow-up MRI showed no signs of development of new lesions or other cerebral conditions. This means that, for this patient, CLOCC has shown persistent complete clinical recovery long-term, and has not yet shown to predispose for, be a precursor to, or otherwise be correlated to any other neurological conditions. A limitation of this study is the absence of formal neurocognitive assessments beyond the patient's self-reports during follow-up. While these could provide insight into the clinical relevance of subtle white matter changes, the lack of baseline assessments prevents reliable attribution of any deviations from normative scores. Additionally, the patient reported no cognitive complaints, which influenced our decision not to conduct further assessments.

Although fitting the main characteristics of previously described CLOCC cases, it stands out for its remarkably short duration of symptoms, as well as radiological changes extending beyond the typical small lesion in the SCC. This adds a novel dimension to our understanding of CLOCC, underscoring the importance of comprehensive case reports in advancing the knowledge of rare neurological conditions. However, the abnormal extent of white matter involvement renders this case an exception, rather than a representation of CLOCC's typical presentation. The relatively modest symptoms despite this extensive lesion are worth pointing out, especially considering the patient was completely asymptomatic at the time the MRI was conducted. Symptoms in CLOCC tend to be correlated to the underlying disease rather than the splenial lesion itself (Moors et al., 2023; Garcia-Monco et al., 2011). There has, to the authors' knowledge, not been proposed any explanations as to why the lesions themselves tend not to cause any symptoms. Follow-up MRI 11 years later displayed some sequela, which is not unheard of for CLOCC, although uncommon (Ueda et al., 2014; Galnares-Olalde et al., 2019). Still, previous cases have only described radiological follow-up periods of a maximum of 10 months (Moors et al., 2023; Galnares-Olalde et al., 2019). Therefore, it is unknown whether these patients would show long-term persistent changes on MRI as described in this case.

The transient neurological symptoms the patient presented with were headache, paresis of the right arm, and dysarthria. Headache is a common symptom in CLOCC (Yum and Shin, 2022). Monoparesis has also previously been described (Tahara et al., 2016; Bulakbasi et al., 2006; Li et al., 2023), even though symptoms being this focal is uncommon. There might be a correlation between this and the MRI changes seen in the corticospinal tract, although the lesion itself has a symmetrical bilateral presentation. Dysarthria has also been reported in several cases (Tetsuka, 2019; Cho et al., 2007; Ryu et al., 2020). MRI showed no obvious reason for dysarthria in our case, so one can only speculate about the cause. One possible explanation is a transient flow reduction in the posterior circulation. However, the radiological findings are not typical for an ischemic injury. Alternatively, the lesion might have led to a transient facial palsy not apparent for the patient herself.

As mentioned previously, several conditions have been reported to be correlated to CLOCC. Pregnancy and infection are relevant to discuss in this case. Most previous cases of CLOCC related to pregnancy report lesions being restricted to the SCC and showing complete resolution on follow-up MRI, with a few exceptions. One case, similarly to ours, describes a postpartum patient presenting with a lesion with extrasplenial involvement that remained hyperintense on FLAIR 90 days later (Ueda et al., 2014). The affected area differs from our case, however, involving the SCC, the genu of the corpus callosum, and the surrounding white matter extending laterally from the SCC (Ueda et al., 2014). The symptoms were also more extensive, including flu-like symptoms, drowsiness, numbness of limbs, delirium, ataxia, right facial palsy, dysphagia, and dysarthria, which disappeared within 1 week, including 3 days of treatment with intravenous corticosteroids and fluid, and reinstitution of a vitamin supplement (Ueda et al., 2014). Another case describes a post-partum psychosis with a splenial lesion extending into the body of the corpus callosum, showing near complete clinical recovery 10 days later, after initiation with antipsychotic medications (Udaya et al., 2015). However, this case has no follow-up imaging (Udaya et al., 2015). Several of the pregnancy-related CLOCC cases report other conditions concurrently with pregnancy/post-partum just before or at the time CLOCC appeared. In our case, the patient's mastitis with systemic affection might have led to inflammatory cytokinopathy in the brain causing excitotoxicity, resulting in CLOCC. One patient has previously been described with a cytotoxic callosal lesion and concurrent mastitis, after presenting with headache and dizziness, but it is uncertain whether this was a genuine CLOCC case due to no follow-up MRI (Barburoglu et al., 2022).

The high signal intensity on DWI and low ADC-values seen in CLOCC, as well as the name “cytotoxic lesions of the corpus callosum,” suggest a cytotoxic nature of these lesions. However, true cytotoxic lesions are typically irreversible. An alternative explanation for the observed reversibility could be intramyelinic edema, a non-neurotoxic fluid confined to the periaxonal space and spaces between the myelin sheaths, which does not cause cell death (de Oliveira et al., 2019; Tetsuka, 2019). This hypothesis is further supported by a recent autopsy case of a patient with CLOCC and hypoglycemia which revealed intramyelinic edema, myelin pallor, loss of fibrous astrocytes, microglial reactions, and minimal lymphocytic infiltration in the parenchyma into the SCC, with intact axons (Hayashi et al., 2023). Presuming intramyelinic edema is the cause of reversibility in the SCC in our case, the partial irreversibility in centrum semiovale may be indicative of cellular death or demyelination, leading to scarring (de Oliveira et al., 2019; Wardlaw et al., 2015). Although our patient shows no overt consequences of this radiological sequela, white matter alterations can subtly impact brain plasticity (De Marco et al., 2017), even in the absence of apparent neurological symptoms. White matter hyperintensities on FLAIR may represent demyelination and FLAIR might even underestimate the extent of demyelination in areas like the centrum semiovale (Haller et al., 2013). Myelin is crucial to efficient signal transmission, and its disruption can impede neuronal conduction. Furthermore, this destruction poses challenges to white matter plasticity, which relies on processes such as new myelin formation, changes in myelin thickness, and internodal length modulation (Sampaio-Baptista and Johansen-Berg, 2017). The persistent hyperintensities observed in the centrum semiovale prompt intriguing questions about their potential impact on white matter plasticity in this case. Despite the asymptomatic nature of our patient, these persistent changes could theoretically influence white matter structural properties and its contributions to neural network functionality. Although the direct clinical significance of these findings remains undetermined in this patient, they underscore the necessity for further research exploring how persistent radiological changes interact with brain plasticity and network dynamics over time. Therefore, highlighting the non-reversible aspects of this CLOCC case is crucial.

## Conclusions

The presented case portrays clinical and radiological findings that align with the established profile of CLOCC, yet introduces a unique perspective to our comprehension of the disorder, emphasizing the crucial role of comprehensive case reports in advancing knowledge of rare neurological conditions. The patient presented 10 days postpartum with unusually brief and focal symptoms. Remarkably, MRI conducted the following day revealed extensive and widespread changes despite the rapid resolution of symptoms, and displayed significant regression 4 days later. In an 11-year follow-up, the longest reported follow-up period for CLOCC, MRI revealed near disappearance of the initial lesion, with no signs of new cerebral lesions or other cerebral conditions. Importantly, the patient has remained free of sequelae or new neurological symptoms since the initial episode. The decade-long follow-up renders the results of this case particularly pertinent for future studies exploring the long-term development and prognosis of CLOCC. Notably, this case highlights the potential for non-reversible white matter changes in CLOCC, challenging the perception of complete recovery. Future research should investigate the subtle, long-term consequences of these changes to better advise on lifestyle modifications that may promote cognitive resilience and brain health later in life.

## Data Availability

The original contributions presented in the study are included in the article/Supplementary material, further inquiries can be directed to the corresponding author.
