# Supplementary material for: Case report: Re-evaluating reversibility of cytotoxic lesions of the corpus callosum
Source: Front Neuroimaging. 2025 Feb 17;4:1436931. doi: 10.3389/fnimg.2025.1436931 (PMC11872946; doi:10.3389/fnimg.2025.1436931)
Supplement: Supplementary file 1 [file Data_Sheet_1.pdf]

SUPPLEMENTARY MATERIAL:

Methods:

Supplementary table: MRI acquisition parameters across time points

	T2TSE TR/TE/Slice thickness(spacing)	DWI TR/TE/mm slice thickness(spacing)	FLAIR TR/TE/TI
1. MRI	4819/100/5(6)	4627/112/5(6)#	6000/120/5(6)
2. MRI	4455/100/5(6)	4666/86/5(6)	6000/100/5(6)
3. MRI	5824/100/4(5)	4732/94,2/4(5)	4800/209/1660*

This table details the MRI acquisition parameters for T2-weighted turbo spin echo (T2TSE), diffusion-weighted imaging (DWI), and fluid-attenuated inversion recovery (FLAIR) sequences, across three different time points in the study. The repetition time (TR), echo time (TE), and slice thickness with spacing are indicated for each of the T2TSE and DWI scans. For FLAIR, also inversion time (TI) is listed. The third MRI scan (denoted by *) utilized a 3D FLAIR approach, in contrast to the 2D FLAIR sequences used in earlier scans. The scans from the third MRI scan were performed using a SENSE-head-8 coil, enhancing image quality and detail.

Supplementary figure:

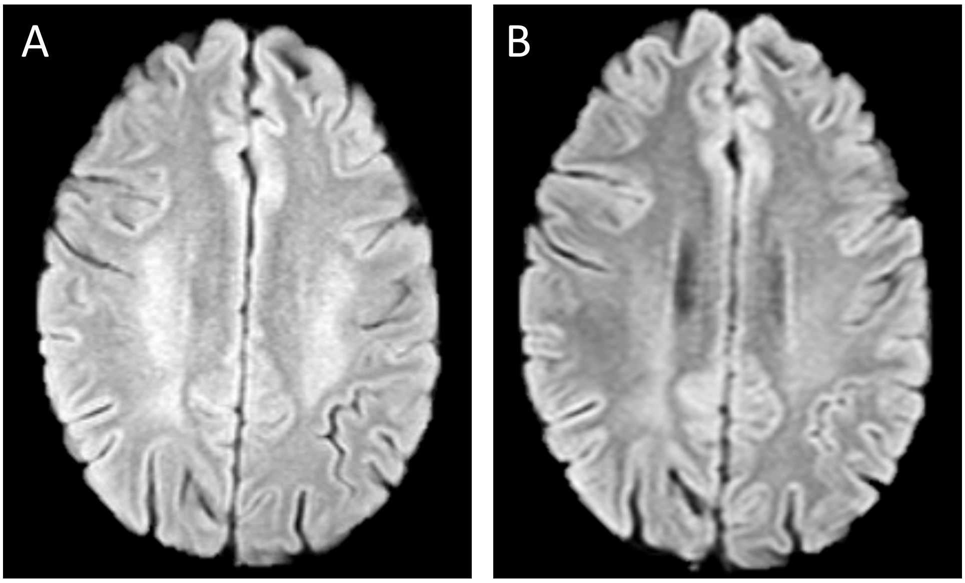

Supplementary figure: Comparison of FLAIR images with adjusted slice thickness

Panels A and B depict FLAIR images from the original MRI scan performed at the time of presentation (Panel A) and the follow-up MRI scan acquired 11 years and 5 months later (Panel B). Both images have been adjusted to a slice thickness of 5 mm with 6 mm spacing to enhance comparability across the time points. Panel A presents the original 2D FLAIR scan, while Panel B displays the 3D FLAIR scan from the follow-up. This adjustment ensures a consistent presentation of structural details in the images, highlighting the persistent radiological findings over the longitudinal interval. The modifications demonstrate that the observed pathology remains visible despite adjustments in slice parameters, supporting the robustness of these findings over time.
